# Comorbid Depression and Diabetes Are Associated with Impaired Health-Related Quality of Life in Chronic Kidney Disease Patients

**DOI:** 10.3390/jcm11164671

**Published:** 2022-08-10

**Authors:** Janine Wirkner, Matthias Scheuch, Thomas Dabers, Sabrina Freiin von Rheinbaben, Beate Fiene, Simone Aymanns, Karlhans Endlich, Nicole Endlich, Uwe Lendeckel, Rainer Rettig, Hans Jörgen Grabe, Sylvia Stracke

**Affiliations:** 1 Clinical Psychology, Institute for Psychology, University of Greifswald, 17489 Greifswald, Germany; 2 Department of Internal Medicine A, Nephrology, University Medicine Greifswald, 17475 Greifswald, Germany; 3 Department of Anatomy and Cell Biology, University Medicine Greifswald, 17475 Greifswald, Germany; 4 Institute of Medical Biochemistry and Molecular Biology, University Medicine Greifswald, 17475 Greifswald, Germany; 5 Institute of Physiology, University Medicine Greifswald, 17475 Greifswald, Germany; 6 Department of Psychiatry and Psychotherapy, University Medicine Greifswald, 17475 Greifswald, Germany

**Keywords:** chronic kidney disease, eGFR, health-related quality of life, depression, diabetes, dialysis

## Abstract

Given the increasing prevalence of chronic kidney disease (CKD) and its impact on health care, it is important to better understand the multiple factors influencing health-related quality of life (HRQOL), particularly since they have been shown to affect CKD outcomes. Determinants of HRQOL as measured by the validated Kidney Disease Quality of Life questionnaire (KDQOL) and the Patient Health Questionnaire depression screener (PHQ-9) were assessed in a routine CKD patient sample, the Greifswald Approach to Individualized Medicine (GANI_MED) renal cohort (N = 160), including a wide range of self-reported data, sociodemographic and laboratory measures. Compared to the general population, CKD patients had lower HRQOL indices. Dialysis was associated with (1) low levels of physical functioning, (2) increased impairments by symptoms and problems, and (3) more effects and burden of kidney disease. HRQOL is seriously affected in CKD patients. However, impairments were found irrespective of eGFR decline and albuminuria. Rather, the comorbid conditions of depression and diabetes predicted a lower HRQOL (physical component score). Further studies should address whether recognizing and treating depression may not only improve HRQOL but also promote survival and lower hospitalization rates of CKD patients.

## 1. Introduction

To date, more than ten percent of the world’s population is affected by chronic kidney disease (CKD) [1]. Given the rising prevalence of risk factors for CKD such as diabetes, hypertension and other cardiovascular disorders and the increasingly older population, an increase in CKD prevalence is expected during the next decades [2,3]. CKD is either defined by structural renal pathology (or kidney transplant), by a reduced estimated glomerular filtration rate (eGFR) < 60 mL/min/1.73 m², and/or by a urinary albumin-to-creatinine ratio (ACR) of >30 mg/g creatinine for at least three months [1].

Multidisciplinary approaches now highlight the significance of CKD not only by examining physiological and laboratory measures, but also by focusing on patients’ health-related quality of life (HRQOL) and the importance of health care [4]. HRQOL describes the well-being in different physical, psychological and social domains of functioning and is well known to be influenced by multiple factors, including clinical manifestation of the disease and side effects of treatment, as well as the quality of interaction with family and health care providers. Moreover, chronic disease predisposes to an increased risk for mental health, especially depression. The reported point prevalence of depressive symptoms in CKD varies widely from 1.4 to 94.4%, depending on study populations, with dialysis patients showing the highest symptom scores [5].

In CKD patients, HRQOL is frequently assessed using the validated Kidney Disease Quality of Life (KDQOL) questionnaire from the RAND Corporation [6,7,8]. The HRQOL in CKD patients has been examined in population-based and in clinical samples using different sociodemographic variables, comorbid conditions and physiological measures as potential risk factors [9]. Being a main criterion in CKD definition, eGFR is one of the best-described physiological parameters in HRQOL research. In previous studies, low eGFR was associated with a decrease in physical and mental HRQOL [2,10,11,12,13]. Besides low eGFR, some studies also reported albuminuria to go along with impaired HRQOL in CKD [12]. Decreasing serum albumin levels over time were also associated with lower HRQOL in CKD 3-5 patients [2] and with impairments in physical functioning in patients on hemodialysis [10]. Moreover, decreased serum hemoglobin levels were associated with impaired HRQOL, and in particular with lower physical and cognitive functioning in non-CKD patients [11,12,14,15]. Regarding sex differences, inconsistent findings have been reported [2,11,14,16]. Finally, other sociodemographic variables such as age and education, as well as the comorbid conditions of obesity, diabetes mellitus and cardiovascular diseases have also been discussed in the context of a reduced HRQOL; however, the results were not consistent [12,14,17]. As conflicting results are found in the literature, it is unclear whether CKD itself might be responsible for lower HRQOL or whether an impaired quality of life might be rather a consequence of certain comorbid conditions which could possibly be treated easier than CKD itself [18,19].

The present study examined HRQOL in CKD patients of different CKD stages and its associations with a wide range of parameters, including sociodemographic and physiological measures, as well as comorbid conditions and dialysis dependency, in a routine CKD patient sample (GANI_MED renal cohort). We expected CKD patients to show impaired HRQOL when compared to the general population. We also hypothesized that CKD (lower eGFR and increasing albuminuria) would not be the main influential parameter when taking other factors like sociodemographic, physiological parameters and comorbid depression into account.

## 2. Materials and Methods

### 2.1. Participants and Study Design

Data from 400 patients of the Greifswald Approach to Individualized Medicine (GANI_MED) renal insufficiency cohort with chronic kidney disease (CKD) were collected between July 2011 and April 2015 from in-hospital patients or patients from dialysis centers. During this time, all consecutive patients with CKD or end-stage renal disease were informed by the heads of the institutions (SS, TD) and were then asked to participate in the study and provided written consent forms (BF, SA). Patients with chronic and end-stage renal failure were included. The exclusion criteria were acute renal failure, acute infection and missing informed consent. A total of 162 patients with CKD returned the Kidney Disease Quality of Life (KDQOL) questionnaire they were asked to complete after the main examination. One patient was excluded from the analysis because of missing laboratory data due to technical reasons and one patient because of a low eGFR category (G1), resulting in a final sample size of 160 (response rate: 40%). Complete Patient Health Questionnaire (PHQ-9) data were available from 132 patients (33%), and full data from 62 patients (15.5%) were available for regression analysis. For cohort profile and detailed study procedures, please refer to Grabe et al. (2014) [20]. GANI_MED was approved by the ethics committee of the Medical Faculty of the University of Greifswald. All individual participants included in the study provided informed written consent. All procedures performed in the present study involving human participants were in accordance with the ethical standards of the institutional research committee and with the 1964 Helsinki declaration and its later amendments or comparable ethical standards.

### 2.2. Kidney Disease Quality of Life Questionnaire

Health-related quality of life (HRQOL) was assessed using the Kidney Disease Quality of Life (KDQOL) questionnaire, short form (KDQOL-SFTM), from the RAND corporation [6,7,21]. The KDQOL includes the SF (Short Form)-36 questionnaire (http://www.sf-36.org (accessed on 15 February 2020)) and additional kidney disease-specific scales (Kidney Disease Component Summary, KDCS). The 36 items of the SF-36 measure eight dimensions of functioning and well-being (physical functioning, role limitations caused by physical problems, pain, general health, vitality, social function, role limitations caused by emotional problems and mental health) [22]. Furthermore, the physical composite summary (PCS) score and the mental composite score (MCS) were calculated using the standard RAND scoring algorithm according to the manual [6]. The KDCS consists of 43 items measuring eleven domains (symptom/problem, effects of kidney disease, burden of kidney disease, work status, cognitive function, quality of social interaction, sexual function, sleep, social support, dialysis staff encouragement and patient satisfaction) and one general health-rating item. All scales except the composite scores (T-values) are given on a 100-point Likert scale, with higher values indicating better quality of life.

### 2.3. Patient Health Questionnaire (PHQ-9)

The PHQ-9 is a self-rating scale out of the Patient Health Questionnaire which scores the nine DSM-IV criteria for depression during the last two weeks and provides a valid dimensional measure of depression severity [23].

### 2.4. Procedures

Patients with CKD first received a complete study description and provided written informed consent. Although not classified as CKD according to eGFR stages, the patients in stages < G3a were initially included because of meeting other criteria for CKD like albuminuria or structural renal pathology. To assess sociodemographic data and medical history, computer-assisted standardized interviews were conducted by trained and certified interviewers, and the data were directly entered into a portable computer [20]. Blood pressure, weight and height were measured using pre-defined standard operating procedures. Moreover, on the day of recruitment, blood and urine samples were obtained from the participants and a standardized set of laboratory parameters was measured. The participants were requested to fill in and return the self-report questionnaires (KDQOL, PHQ-9) after completing the main examination.

### 2.5. Statistical Methods

All analyses were conducted using Stata^®^ 13 SE Data Analysis and Statistical Software (StataCorp LP, College Station, TX, USA). Univariate ANOVAs and Chi² or Fisher’s exact test (cell frequency < 5) were calculated for parametrical and non-parametrical testing, respectively. For post hoc comparisons, Bonferroni-corrected p-values are reported. Two-sample *t*-tests were calculated to compare SF-36 scales of the study population with the general population. For statistical relationship testing, pairwise correlations were calculated. To test the association between the physical and mental composite summary scores and age, gender, marital status and retirement, dialysis, BMI, depression and laboratory parameters (eGFR, albuminuria, hemoglobin, HbA1c), multiple linear regression analyses were conducted (controlled for multicollinearity).

## 3. Results

### 3.1. Sociodemographics and Comorbid Conditions

Sociodemographics and comorbid conditions are shown in Table 1. A total of 114 patients were in the eGFR category G5/G5D, 16 in G4, 14 in G3b, 7 in G3a, and 9 in G2, respectively. All patients were Caucasian. The patients in different eGFR categories did not differ significantly with respect to age (*p* = 0.124), gender distribution (*p* = 0.811), education (*p* = 0.988) and marital status (*p* = 0.827). Patients in higher eGFR categories were more often retired (*p* < 0.001). The frequency of comorbid conditions did not differ among eGFR categories.

### 3.2. Physiological Parameters and Laboratory Measures

The mean BMI (29.6 kg/m²) indicated overweight in the present sample and was comparable between eGFR categories (*p* = 0.422; see Table 2). Systolic blood pressure (*p* = 0.220) did not differ significantly among groups, but diastolic blood pressure was significantly lower in G5/G5D (*p* < 0.001). Serum creatinine was highest in eGFR category G5/G5D compared to all other categories (*p* < 0.001). The serum levels of calcium (*p* = 0.562), hemoglobin (*p* = 0.409) and urinary albumin-to-creatinine ratio (UACR; *p* = 0.112) did not differ significantly among the groups, whereas the serum levels of phosphorus were significantly higher in G5/G5D than in the other categories (all *p* < 0.001). The patients in category G2 had higher albuminuria than the patients in G5/G5D (*p* = 0.009). The serum levels of HbA1c were significantly higher in G3a, G3b and G4 compared to G2 and G5/G5D. Serum parathyroid hormone concentration could not be analyzed statistically due to missing distributions between CKD stages.

### 3.3. Health-Related Quality of Life

CKD patients had lower SF-36 scores than the general population (SD-36 norms, all *p* < 0.001, see Table 3). Moreover, PCS was about two standard deviations lower than the mean (T-value = 32.0), a finding that was not true for the MCS (T-value = 47.1), showing that physical condition was significantly impaired in CKD patients while mental health was in the normative range. No eGFR group differences were found for the SF-36 dimensions (physical functioning: *p* = 0.118; role physical: *p* = 0.665; pain: *p* = 0.536; general health: *p* = 0.329; vitality: *p* = 0.514; social functioning: *p* = 0.563; role emotional: *p* = 0.865; mental health: *p* = 0.657) and the physical (*p* = 0.236) and mental composite summary scores (*p* = 0.826). Women had lower levels of social functioning (p = 0.027) than men.

The kidney disease targeted areas work status (*p* = 0.358), cognitive function (*p* = 0.730), quality of social interaction (*p* = 0.682), sexual function (*p* = 0.105), sleep (*p* = 0.361) and social support (*p* = 0.251) did not differ significantly among the eGFR categories (Table 4). Dialysis staff encouragement and patient satisfaction could not be analyzed statistically due to missing data in various CKD stages. However, kidney disease symptom/problem was elevated in G2 and G3a patients compared to others (*p* = 0.047). The effects of kidney disease were higher in G5/G5D patients than in G4 patients (*p* < 0.001), and the burden of kidney disease was higher in G5/G5D compared to patients in other categories (*p* < 0.001). Men reported higher impairments in sexual functioning than women (*p* < 0.001). PHQ-9 summary scores did not differ among the eGFR categories (*p* = 0.226).

In the CKD sample, 21 patients (13.1%) had moderate depression, 9 (5.6%) had moderately severe depression and 19 (12.0%) had severe depression, according to PHQ-9 summary scores, indicating that as much as 30.7% of all CKD patients showed depressive symptomatology (PHQ-9 ≥ 10; Manea et al., 2012).

Patients on dialysis had significantly lower levels of physical functioning and a decreased PCS score compared to non-dialysis patients. Moreover, dialysis resulted in worse symptom/problem ratings, effects and the burden of kidney disease, and sexual function scores (see Table 5).

Using pairwise correlations, a higher eGFR was associated with a better physical component summary (r = 0.168, *p* = 0.049) as well as with the effects of kidney disease (r = 0.295, *p* < 0.001) and the burden of kidney disease (r = 0.342, *p* < 0.001) scores. Surprisingly, a higher UACR was associated with better physical functioning (r = 0.248, *p* = 0.010), vitality (0.214, *p* = 0.031), social functioning (0.232, *p* = 0.016) and symptom/problem scores (r = 0.194, *p* = 0.049). HbA1C was related to physical component summary scores only (r = −0.169, *p* = 0.049), and serum hemoglobin was associated with social support (r = −0.194, *p* = 0.015). Serum phosphorus and calcium levels and systolic blood pressure were not statistically associated with SF-36 measures, but higher diastolic blood pressure were associated with better physical functioning (r = 0.304, *p* < 0.001), pain (r = 237, *p* = 0.003), general health perception (r = 0.169, *p* = 0.068), and physical component summary score ratings (r = 0.288, *p* = 0.001), as well as in better symptom/ problem (r= 0.233, p= 0.003), effects of kidney disease (r = 0.227, *p* = 0.006), sexual functioning (r = 0.186, *p* = 0.042) and sleep (r = 0.180, *p* = 024) ratings. A higher BMI was associated with worse physical functioning (r= −0.199, *p* = 0.015), pain (−0.199, *p* = 0.015) and physical component summary scores (r = −0.199, *p* = 0.032).

Multiple linear regression analysis revealed a significant negative influence of higher age on PCS scores (see Table 6 for SF-36 analyses and regression coefficients). Interestingly, only depression severity was associated with lower physical and mental composite summary scores, and HbA1C was related to worse PCS, but better MCS. No significant associations between renal function (eGFR, albuminuria) and health-related quality of life were observed when controlling for the other predictors.

## 4. Discussion

It is known that HRQOL of renal patients is low, especially in the physical component score. There is, however, limited literature on treatable conditions in the low HRQOL of CKD patients. Here, we show for the first time that diabetes and depression are independent predictors of PCS and MCS in patients with CKD; both are conditions that are possible treatment targets.

While for a long time research on HRQOL focused on end-stage renal disease, in our cohort, we analyzed both dialysis and non-dialysis CKD patients. In the present study, CKD patients of all stages had lower HRQOL scores than the general population, except for the mental component summary score. Regarding the component summary scores, the literature reports are quite consistent over different study populations [2,10,24], showing lower PCS in CKD patients compared to the general population and an MCS that is still within the normative range. Replicating previous findings, physical impairments were the most burdensome [11,25,26]. Overall, up to one-quarter of CKD patients are supposed to suffer from depressive symptoms [5,27]. With a mean point prevalence of 20%, depressive symptomatology in the present CKD sample was twice as high as in the general German population [28]. The small sample size of our study, however, does not allow a general conclusion.

At first glance, a higher eGFR was associated with better physical functioning, general health perception, PCS, symptom/ problem, effects and burden of kidney disease scores, as well as with work status, suggesting that low renal function by itself might be responsible for impaired HRQOL. Indeed, previous studies reported associations between renal function and physical or mental HRQOL [2,10,11,12]. Lower eGFR and increased albuminuria have both been found to be associated with impaired HRQOL in CKD [2,10,12]. However, in the present study, when controlling for various sociodemographic and physiological parameters and for comorbidity, this association remained no longer statistically significant. In line, Campbell et al. (2013) [17] found that, after adjustment for demographics and comorbid conditions, eGFR was no longer related to physical or mental HRQOL in outpatients with diabetes mellitus, regardless of the presence or absence of proteinuria [7]. Moreover, these authors reported a strong negative association between eGFR and the risk of depressive symptoms, highlighting the need for addressing depression in this population. Thus, as kidney disease itself shows no clear symptoms, and eGFR is not independently related to HRQOL measures, other influential factors such as depression might contribute to quality of life instead. Depressive symptoms in CKD have been associated with an increased risk of hospitalization, dialysis initiation and mortality [29,30]. Additionally, in a longitudinal study with 3837 CKD patients of the CRIC cohort, in fully adjusted models, low physical component summary scores were independently associated with a higher risk of cardiovascular events and death, whereas low mental component summary scores were independently associated with a higher risk of death [18]. However, as we also found in our GANI_MED renal cohort, low HRQOL was not associated with CKD stages. In contrast, depression predicted both low physical and low mental component scores, suggesting that it is essential to diagnose and treat depression in CKD patients [30,31]. However, antidepressants might not be effective in CKD (for a critical discussion of the role of antidepressants in the treatment of depression, see Kirsch et al., 2011) [32]. The “CKD Antidepressant Sertraline Trial” showed no benefit of a serotonin-selective reuptake inhibitor (SSRI), sertraline, over a double-blind matched placebo for the treatment of depressive symptoms in patients with non-dialysis CKD [33]. In line with Zalai et al. (2012), we suggest that time-limited manualized psychotherapies, e.g., cognitive-behavioral approaches, should be provided to CKD patients with clinically relevant depression [34].

In the present study, the patients on dialysis did not differ in depression scores from the non-dialysis patients. However, the dialysis patients showed worse levels of physical functioning, symptoms/problems, the effects and burden of kidney disease and sexual function, corroborating previous findings [18,24]. The associations of progressive CKD and dialysis with impairments in self-reported cognitive functioning were not evident, although cognitive impairments have been reported in patients on renal replacement therapy and pre-dialysis [35,36]. Perhaps, KDQOL self-report items have only limited sensitivity to measure actual cognitive decline [37]. Sexual functioning also worsened with increasing comorbid conditions, with female patients indicating less impairment than men. Unfortunately, sexual dysfunction is still an under-recognized burden in CKD patients and clinical trials of treatment options are scarce [38,39].

Among all eGFR categories, the CKD G2 patients showed the highest albuminuria levels. Usually, CKD G1 and G2 are not clinically relevant unless albuminuria is found. This might explain the high ACR in this group.

Higher Hb1Ac levels as a marker for average long-term blood glucose levels and diabetes mellitus were associated with lower PCS. This is in line with previous findings reporting worse HRQOL outcomes in comorbid diabetic patients [2,40]. In order to improve HRQOL and clinical outcomes, enhancing physical functioning by treating diabetes might be crucial in CKD patients, especially in stage 3b [11,33]. Unfortunately, although early and recent research has linked hyperparathyroidism to affective and cognitive symptoms, parathyroid hormone levels have not yet gained sufficient attention in CKD patients with depression [37,41].

The limitations of our study are the response bias and missing data. A total of 162 out of 400 patients responded to the questionnaire. The final regression analysis involved data from only 62 patients. The results of this study need further research and confirmation.

## 5. Conclusions

In the present study, CKD patients showed impairments in quality of life, a finding that was especially true for dialysis patients. In the GANI_MED patient sample drawn from routine hospital patients and patients from dialysis centers, renal function assessed by eGFR and albuminuria was not independently associated with HRQOL measures. Instead, depression severity accounted for both impaired physical and mental HRQOL, and diabetes was associated with lower physical composite summary scores. Further studies should address whether recognizing and treating depression may not only improve HRQOL but also promote survival and lower hospitalization rates of CKD patients.

## Figures and Tables

**Table 1:** Sociodemographic data and comorbid conditions.

		eGFR Category				
	All	G2	G3a	G3b	G4	G5/G5D
n	160	9	7	14	16	114
Mean age (SD) [years]	66.6 (14.2)	55.7 (19.8)	68.3 (8.5)	63.4 (13.5)	68.0 (15.4)	67.6 (13.6)
Male	99 (61.9)	5 (55.6)	5 (71.4)	7 (50.0)	11 (68.8)	71 (62.3)
Education [years]						
n = 149						
<8	2 (1.3)	0 (0)	0 (0)	0 (0)	0 (0)	2 (1.8)
8	81 (54.4)	3 (42.9)	2 (33.3)	6 (54.5)	8 (61.5)	62 (55.4)
10	45 (30.2)	3 (42.9)	3 (50.0)	4 (36.4)	4 (30.8)	31 (27.7)
12	21 (14.1)	1 (14.3)	1 (16.7)	1 (9.1)	1 (7.7)	17 (15.2)
Married	98 (61.2)	5 (55.6)	4 (57.1)	8 (57.1)	8 (50.0)	73 (64.0)
Retired n = 125	110 (88.0)	3 (50.0)	3 (100.0)	3 (50.0)	5 (83.3)	96 (92.3)
Dialysis	109 (68.1)	0 (0)	0 (0.0)	0 (0.0)	0 (0.0)	109 (95.6)
Diabetes mellitus n = 159	78 (49.1)	1 (12.5)	5 (71.4)	9 (64.3)	5 (31.3)	58 (50.9)
Hypertension	139 (86.9)	7 (77.8)	6 (85.7)	11 (78.6)	12 (75.0)	103 (90.4)
Peripheral artery disease n = 156	34 (21.8)	1 (11.1)	0 (0.0)	5 (35.7)	3 (18.8)	25 (22.7)
Atrial fibrillation n = 142	33 (23.2)	0 (0)	2 (33.3)	1 (8.3)	2 (13.3)	28 (28.0)
Myocardial infarction n = 158	30 (19.0)	1 (11.1)	2 (28.6)	5 (35.7)	2 (13.3)	20 (17.7)
Angina pectoris n = 159	37 (23.3)	1 (11.1)	0 (0)	3 (21.4)	7 (43.8)	26 (23.0)
Stroke n = 157	20 (12.8)	0 (0)	0 (0)	2 (14.3)	1 (6.3)	17 (15.2)
Cancer n = 156	34 (21.4)	1 (12.5)	0 (0)	3 (21.4)	5 (31.3)	25 (22.1)
Osteoporosis n = 147	21 (14.4)	0 (0)	0 (0)	2 (16.7)	3 (21.4)	16 (14.5)

Values represent absolute numbers (%) except for age.

**Table 2:** Physiological parameters and laboratory measures.

		eGFR Category				
	All	G2	G3a	G3b	G4	G5/G5D
Mean BMI [kg/m^2^] n = 151	29.6 (6.3)	27.1 (5.6)	31.5 (6.1)	31.8 (8.3)	28.6 (5.2)	29.5 (6.2)
Systolic blood pressure [mmHg] n = 158	130.8 (19.9)	122.2 (14.5)	138.5 (30.9)	137.9 (23.0)	135.4 (20.4)	129.5 (18.8)
Diastolic blood pressure [mmHg] n = 158	72.8 (10.6)	77.8 (6.7)	70.3 (10.4)	81.1 (15.2)	77.7 (9.6)	70.9 (9.6)
Mean eGFR [ml/min/1.73m^2^] n = 160	19.1 (18.0)	70.5 (9.1)	50.9 (4.8)	36.8 (4.5)	22.4 (4.8)	10.5 (7.3)
Serum creatinine [µmol/l] n = 160	434.6 (250.7)	88.3 (15.3)	114.7 (8.7)	148.4 (29.2)	239.3 (43.7)	544.1 (211.1)
Serum calcium [mmol/L] n = 159	2.2 (0.19)	2.1 (0.20)	2.2 (0.16)	2.2 (0.15)	2.2 (0.43)	2.2 (0.15)
Serum phosphorus [mmol/l] n = 159	1.3 (0.49)	1.0 (0.26)	0.92 (0.19)	1.2 (0.31)	1.1 (0.28)	145 (0.52)
Urine albumin [mg/L] n = 108	725.1 (1488.8)	2418.9 (3157.2)	789.9 (1444.9)	763.5 (1085.7)	1197.2 (2762.8)	440.2 (597.1)
Urinary albumin-to-creatinine ratio n = 105	994.3 (1741.8)	2489.4 (3355.5)	1187.5 (2424.2)	831.7 (1047.3)	1417.8 (2829.0)	751.3 (1075.5)
Serum hemoglobin [mmol/L] n = 158	7.0 (1.0)	7.6 (1.8)	7.2 (1.3)	7.1 (1.4)	7.3 (0.97)	6.9 (0.91)
Serum HbA1c [%] n = 158	6.0 (1.2)	5.7 (1.1)	6.3 (0.7)	6.4 (1.2)	6.7 (1.1)	5.8 (1.1)
Serum parathyroid hormone (ng/L) n = 79	254.0 (179.9)	-	-	53.0 (42.4)	-	259.2 (179.2)

Values represent means (SD).

**Table 3:** SF-36 dimensions of functioning and composite scores.

		eGFR Category					
	All	G2	G3a	G3b	G4	G5/G5D	General Population *
Physical functioning n = 157	38.7 (30.3)	56.3 (27.2)	53.6 (31.1)	41.8 (26.9)	45.6 (32.8)	35.0 (30.1)	84.2 (23.3)
Role physical n = 153	25.1 (36.3)	25.0 (43.3)	32.1 (47.2)	13.5 (28.2)	18.3 (34.7)	27.0 (36.3)	81.0 (34.0)
Pain n = 157	53.0 (31.6)	63.3 (33.9)	61.1 (33.2)	60.7 (32.7)	46.4 (33.2)	51.5 (31.1)	75.2 (23.7)
General health n = 152	40.3 (18.5)	47.9 (13.1)	43.7 (21.1)	47.5 (14.8)	40.0 (12.7)	38.6 (19.6)	72.0 (20.3)
Vitality n = 151	43.0 (20.0)	50.6 (27.0)	48.6 (17.5)	37.5 (15.6)	46.0 (18.5)	42.1 (20.1)	60.9 (21.0)
Social functioning n = 159	66.1 (28.1)	73.6 (28.9)	76.8 (15.2)	72.3 (27.8)	61.7 (28.3)	64.7 (28.1)	83.3 (22.7)
Role emotional n = 150	47.7 (48.0)	48.1 (50.3)	47.6 (50.4)	35.9 (48.0)	55.3 (48.3)	47.8 (48.2)	81.3 (33.0)
Mental health n = 149	67.5 (19.4)	69.7 (18.8)	72.6 (18.7)	60.3 (22.4)	69.8 (15.8)	67.5 (19.7)	74.7 (18.1)
PCS [T-value] n = 138	32.0 (10.3)	36.7 (9.3)	35.8 (11.7)	35.9 (10.9)	31.7 (10.5)	30.9 (10.2)	
MCS [T-value] n = 139	47.1 (11.6)	47.4 (10.7)	48.4 (10.1)	43.2 (13.8)	48.4 (10.5)	47.2 (11.6)	

Values represent means (SD), and SF-36 summary scales range from 0 to 100, with higher numbers indicating better quality of life (PCS = physical composite summary; MCS = mental composite summary). * Data from the SF-36 manual (n = 2474).

**Table 4:** Kidney disease targeted areas (KDQOL) and PHQ-9.

		eGFR Category				
	All	G2	G3a	G3b	G4	G5/G5D
Symptom/problem n = 158	73.0 (16.5)	85.2 (8.4)	84.2 (7.8)	72.7 (12.8)	73.9 (15.8)	71.2 (17.3)
Effects of kidney disease n = 149	62.8 (22.9)	80.2 (17.9)	85.0 (15.2)	71.6 (20.1)	81.3 (18.9)	57.3 (21.8)
Burden of kidney disease n = 154	48.0 (26.0)	65.2 (26.7)	83.3 (16.1)	63.9 (25.8)	62.9 (25.7)	41.2 (23.0)
Work status n = 155	23.2 (32.4)	33.3 (43.3)	35.7 (37.5)	34.6 (37.6)	18.8 (35.9)	20.9 (29.8)
Cognitive function n = 157	79.3 (17.2)	75.6 (18.0)	85.7 (16.1)	78.6 (17.8)	82.5 (12.1)	78.8 (17.9)
Quality of social interaction n = 158	82.5 (15.8)	81.5 (21.0)	81.9 (19.5)	79.0 (19.7)	87.5 (10.6)	82.5 (15.8)
Sexual function n = 122	54.8 (38.0)	71.9 (34.6)	43.8 (51.5)	75.0 (30.1)	65.6 (43.7)	49.7 (36.9)
Sleep n = 158	61.2 (21.5)	73.1 (11.4)	52.1 (30.0)	58.0 (22.4)	62.8 (15.8)	60.9 (22.1)
Social support n = 158	70.9 (23.0)	59.3 (31.3)	78.6 (15.9)	75.0 (23.3)	78.1 (24.1)	69.8 (22.2)
Dialysis staff encouragement n = 113	83.8 (16.3)	100 (0.0)	100 (-)	75.0 (-)	75.0 (-)	83.6 (16.4)
Patient satisfaction n = 115	68.8 (18.5)	66.7 (23.6)	75.0 (35.5)	50.0 (-)	50.0 (-)	69.1 (18.4)
PHQ-9 summary score n = 131	6.2 (4.8)	6.4 (4.2)	4.3 (2.5)	7.6 (4.8)	3.1 (34.4)	6.4 (5.0)

Values represent means (SD). KDQOL scales range from 0 to 100, with higher numbers indicating better QOL. PHQ-9 ranges from 0 to 27, with higher numbers indicating higher depression severity.

**Table 5:** HRQOL for patients with and without dialysis.

	Dialysis		Analysis	
	Yes	No	F	*p*
Physical functioning	34.13 (29.93)	49.24 (29.52)	**8.97**	**0.003**
Role physical	27.32 (36.56)	21.5 (36.07)	<1	0.354
Pain	51.21 (31.06)	57.02 (32.42)	1.18	0.280
General health	38.53 (19.63)	44.39 (15.52)	3.42	0.066
Vitality	41.71 (20.16)	45.61 (19.30)	1.27	0.261
Social functioning	64.58 (29.03)	69.71 (26.00)	1.17	0.281
Role emotional	47.17 (48.09)	49.67 (48.25)	<1	0.763
Mental health	67.35 (19.91)	68.06 (18.41)	<1	0.835
PCS	30.76 (10.22)	34.86 (10.28)	**4.95**	**0.028**
MCS	47.10 (1.87)	47.13 (10.94)	<1	0.991
Symptom/problem	71.12 (17.51)	77.30 (13.50)	**5.01**	**0.026**
Effects of kidney disease	56.59 (21.75)	78.25 (18.65)	**33.9**	**<0.001**
Burden of kidney disease	41.42 (22.96)	63.30 (26.18)	**27.29**	**<0.001**
Work status	20.48 (29.17)	30.39 (38.83)	3.17	0.077
Cognitive function	79.09 (17.71)	80.00 (16.22)	<1	0.755
Quality of social interaction	82.18 (15.26)	82.82 (17.13)	<1	0.812
Sexual function	48.02 (37.03)	69.51 (36.34)	**9.32**	**0.003**
Sleep	61.02 (22.06)	61.71 (20.44)	<1	0.849
Social support	69.29 (22.34)	74.18 (12.88)	1.59	0.209
PHQ-9 summary score	6.4 (5.0)	5.7 (4.4)	<1	0.438

Values represent means (SD). PCS = physical composite summary; MCS = mental composite summary. Bold highlights *p*-values < 0.05.

**Table 6:** Predictors of health-related quality of life.

N = 62	Physical Composite Summary Score		Mental Composite Summary Score	
Analysis	F (10,51) = 5.73, *p* = 0.000, R² = 0.5292, Adj R² = 0.437		F (10,51) = 4.82, *p* = 0.000, R² = 0.486, Adj R² = 0.385	
Predictors	b	*p*	b	*p*
Age	**−0.24**	**0.010**	−0.05	0.575
Female gender	−3.54	0.142	4.3	0.104
Married	−2.81	0.227	2.14	0.400
Retired	2.81	0.534	−0.34	0.945
Depression severity	**−4.11**	**<0.001**	**−6.29**	**<0.001**
BMI	−0.30	0.094	0.17	0.396
eGFR	−0.04	0.525	−0.04	0.593
Urine albumin (UACR)	0.00	0.514	0.00	0.409
Hb	0.81	0.508	−0.67	0.468
HbA1c	**−2.31**	**0.027**	**2.31**	**0.042**
